# TEAD Proteins Associate With DNA Repair Proteins to Facilitate Cellular Recovery From DNA Damage

**DOI:** 10.1016/j.mcpro.2023.100496

**Published:** 2023-01-12

**Authors:** Philamer C. Calses, Victoria C. Pham, Alissa D. Guarnaccia, Meena Choi, Erik Verschueren, Sietske T. Bakker, Trang H. Pham, Trent Hinkle, Chad Liu, Matthew T. Chang, Noelyn Kljavin, Corey Bakalarski, Benjamin Haley, Jianing Zou, Cuicui Yan, Xia Song, Xiaoyan Lin, Rebecca Rowntree, Alan Ashworth, Anwesha Dey, Jennie R. Lill

**Affiliations:** 1 Departments of Discovery Oncology, Genentech Inc, South San Francisco, California, USA; 2 Department of Microchemistry, Proteomics & Lipidomics, Genentech Inc, South San Francisco, California, USA; 3 UCSF Helen Diller Family Comprehensive Cancer Center, University of California, San Francisco, California, USA; 4 Department of Bioinformatics, Genentech Inc, South San Francisco, California, USA; 5 Department of Molecular Oncology, Genentech Inc, South San Francisco, California, USA; 6 Department of Biology, Research Service Division, WuXi AppTec, Shanghai, China

**Keywords:** TEAD, Hippo pathway, transcription, DNA damage, AP-MS, γH2AX, histone H2AX phosphorylated on S139, γIR, γ-irradiation, AP-MS, affinity purification mass spectrometry, ATM, ataxia telangiectasia mutated, BLM, bleomycin, DSB, double-stranded break, Gy, Gray unit, HR, homologous recombination, NHEJ, nonhomologous end joining, PARP1, poly(ADP-ribose) polymerase 1, NLS, nuclear localization signal, PRKDC, DNA-dependent protein kinase, catalytic subunit, PSM, peptide spectral match, RIF1, Rap1-interacting factor 1, shNTC, nontargeting shRNA control, shRNA, short hairpin RNA, siRNA, small interfering RNA, TAZ, transcriptional co-activator with PDZ-binding motif, TEADs, TEA domain family members 1 to 4, XRCC6, X-ray repair cross-complementing protein 6, XRCC5, X-ray repair cross-complementing protein 5, YAP, Yes-associated protein

## Abstract

Transcriptional enhanced associate domain family members 1 to 4 (TEADs) are a family of four transcription factors and the major transcriptional effectors of the Hippo pathway. In order to activate transcription, TEADs rely on interactions with other proteins, such as the transcriptional effectors Yes-associated protein and transcriptional co-activator with PDZ-binding motif. Nuclear protein interactions involving TEADs influence the transcriptional regulation of genes involved in cell growth, tissue homeostasis, and tumorigenesis. Clearly, protein interactions for TEADs are functionally important, but the full repertoire of TEAD interaction partners remains unknown. Here, we employed an affinity purification mass spectrometry approach to identify nuclear interacting partners of TEADs. We performed affinity purification mass spectrometry experiment in parallel in two different cell types and compared a wildtype TEAD bait protein to a nuclear localization sequence mutant that does not localize to the nucleus. We quantified the results using SAINT analysis and found a significant enrichment of proteins linked to DNA damage including X-ray repair cross-complementing protein 5 (XRCC5), X-ray repair cross-complementing protein 6 (XRCC6), poly(ADP-ribose) polymerase 1 (PARP1), and Rap1-interacting factor 1 (RIF1). In cellular assays, we found that TEADs co-localize with DNA damage–induced nuclear foci marked by histone H2AX phosphorylated on S139 (γH2AX) and Rap1-interacting factor 1. We also found that depletion of TEAD proteins makes cells more susceptible to DNA damage by various agents and that depletion of TEADs promotes genomic instability. Additionally, depleting TEADs dampens the efficiency of DNA double-stranded break repair in reporter assays. Our results connect TEADs to DNA damage response processes, positioning DNA damage as an important avenue for further research of TEAD proteins.

## Highlights

• AP-MS experiments identify DNA damage proteins as interaction partners for TEADs.
• TEADs co-localize with DNA damage-induced nuclear foci marked by γH2AX and RIF1.
• Depleting TEADs by RNAi sensitizes cells to DNA damage and genomic instability.

## Graphical Abstract

**Figure:**
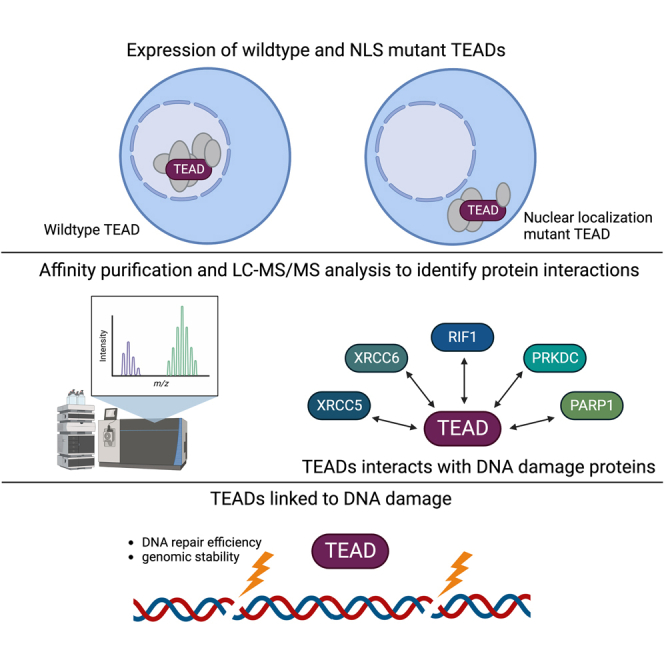

## In Brief

This manuscript describes an affinity purification mass spectrometry approach to evaluate the protein interactions of transcriptional enhanced associate domain family member (TEAD) transcription factors and identifies DNA damage-associated proteins as nuclear interacting partners for TEADs. Follow-up validation experiments with biochemical and cellular assays finds that TEADs colocalize with DNA damage-induced foci and that depletion of TEADs in cells decreases DNA repair efficiencies and promotes genomic instability. These experiments indicate a connection between TEAD transcription factors and DNA repair processes.

Transcription factors are the gatekeepers of gene expression. Aberrant expression or deregulation of transcription factors contributes to human diseases including cancer (1). The transcriptional enhanced associate domain family members 1 to 4 (TEADs) are a set of four transcription factor paralogs that are highly conserved and ubiquitously expressed in higher order organisms. The domain architecture of TEADs is comprised of a DNA binding domain (TEA domain) at the N terminus and a cofactor-binding domain at the C terminus. The N-terminal domain directly interacts with the major and minor grooves of DNA (2) and contains a nuclear localization sequence (NLS) (3). The C-terminal domain mediates interaction with co-activating proteins such as Yes-associated protein (YAP), and its paralog, transcriptional co-activator with PDZ-binding motif (TAZ) (4, 5). Importantly, protein interactions are central to the cellular functions of TEADs.

TEADs function as the transcriptional effectors of the Hippo pathway (6) which is a highly conserved kinase cascade that maintains organ growth and tissue homeostasis (7). Activation of this pathway is dependent on two serine/threonine kinases, mammalian STE20-like 1/2 and large tumor suppressor 1/2, that phosphorylate and negatively regulate YAP and TAZ. In the cytoplasm, phosphorylated YAP and TAZ are sequestered by binding to 14-3-3 and subsequently degraded by the proteasome (8). When the upstream kinases of the Hippo pathway are inactive, YAP and TAZ remain unphosphorylated which allows them to translocate to the nucleus where they directly interact with TEADs to mediate the transcription of genes involved in cellular proliferation, homeostasis, polarity, survival, and cell fate (8). In addition to YAP and TAZ, TEADs are known to engage with other protein binding partners in the nucleus. TEADs interact with the vestigial-like family (VGLL1-4) of transcriptional coactivators, and these interactions can be transcriptionally activating or repressive depending on the context (9, 10, 11). TEADs also interact with AP-1 transcription factors (12, 13) and with chromatin remodeling complexes (14). Clearly the repertoire of TEAD interactions is heavily networked and influences various cellular processes, the full extent of which remains unclear.

The Hippo pathway repeatedly emerges as relevant during cancer development, progression, and therapy resistance (8, 15, 16). A prominent example is the frequent amplification of the effector proteins, YAP, TAZ, and TEADs in squamous, head and neck, and gastrointestinal malignancies (6, 15, 17). Additionally, inducing YAP expression transforms normal epithelial cells to metastatic cells (18) and plays an important role in bypassing RAS signaling in pancreatic and colon cancer progression (19, 20, 21). Although significant progress is being made in elucidating how other signaling pathways can regulate YAP and TAZ in cancer, less is understood about how TEADs are regulated. Moreover, efforts to target the Hippo pathway therapeutically are well underway, in particular, the development of TEAD-targeting small molecules aimed at disrupting the protein interactions that are necessary for TEADs transcriptional function (22, 23). Therefore, understanding the full repertoire of the cellular functions of TEADs will be critical in anticipating the efficacy and potential toxicity of these approaches.

Here, we describe a connection between TEADs and the DNA damage response (DDR) pathway. We performed protein–protein interaction studies using affinity purification mass spectrometry (AP-MS) and found that TEADs interact with several factors known to be involved in DNA double-stranded break (DSB) repair. We found that TEADs colocalize with nuclear DNA damage foci, and that RNAi knockdown of TEADs decreased cellular repair of DSBs and promoted genomic instability. The proteomic and cellular studies presented here provide a rich interaction dataset and describe a connection between the TEAD transcription factors and the crucial cellular response to genomic insults.

## Experimental Procedures

### Experimental Design and Statistical Rationale

The control, WT TEAD3-Myc/Flag, and NLS mutant TEAD3-Myc/Flag (one control, two baits) immunoprecipitations were performed using isotype IgG and anti-Myc Ab, respectively, in HEK293 cell lysates as described in “Cellular fractionation, Western blotting, and Immunoprecipitation'' section below. We carried out the analysis in three biological replicates where each set of control and bait IPs were collected simultaneously to minimize the difference in background proteins between the control and bait IPs. Each biological replicate was done on a different batch of cells. The IP and subsequent mass spectrometric workflow were performed on different days. Similarly, we applied the same strategy for the experiments in Detroit 562 cell lysates. For this analysis, we used the GeLC-MS approach to maximize the chance of detecting the low abundant interactors. Therefore, for practical reasons, we did not perform technical replicates. In addition, our lab has witnessed a high level of reproducibility within technical replicates.

### Software

Figure schemes were created using BioRender.com. Graphs were created using R or using Graphpad Prism. Flow cytometry data were analyzed using FlowJo.

### Cell Lines

HEK293, Detroit 562 (pharyngeal carcinoma cells), MDA-MB-231, and SK-N-F1 (neuroblastoma cells) were cultured in DMEM or RPMI supplemented with 2 mM glutamine, 50 U/ml penicillin, 50 μg/ml streptomycin, 10% heat-inactivated fetal calf serum at 37 °C, and 5% CO_2_. All cell lines described in this study were sourced from ATCC. To generate stable lines used in our experiments, MYC-FLAG-tagged WT TEAD3 cDNA (OHu08942, GeneScript) was first subcloned into PiggyBac (pBind, Genentech) plasmid containing a puromycin resistance and doxycycline inducible cassette by LakePharma. The WT-TEAD3 plasmid was subsequently mutated for seven point mutations to generate the NLS mutant TEAD3 (R87N, R88N, R99N, R100N, K101N, V102N, and R103N) (LakePharma). Stable cell lines were generated by co-transfection of these plasmids with the transposase vector (pBO, Transposagen Biopharmaceuticals) using TransIT-LT1 (Mirus, MIR2300). Transfected cells were then selected with Puromycin (P9620, Millipore-Sigma) at 2 μg/ml. DNA damage in cells was induced by exposing the cells to 6 Gy γ-irradiation (γIR) or treatment with the indicated DNA damaging agents (ex. etoposide, bleomycin).

### Antibodies

Antibodies used for Western blotting: YAP [D8H1X (#14074) or 1A12 (#12395)], TAZ [D3I6D (#70148) or E5P2N (#71192)], Myc-tag [9B11 (#2276) or 71D10 (#2278)], panTEAD [D3F7L (#13295)], Rap1-interacting factor 1 (RIF1) [D2F2M (#95558)], GAPDH [D16H11 (#5174)], α-Tubulin [DM1A (#3873)], pS139 H2A.X [20E3 (#9718)], Ku70 [D10A7 (#4588)], and Ku80 (#2753) from Cell Signaling Technology. MAX [(H2) sc-8011] from Santa Cruz Biotechnology. Secondary antibodies: Goat anti-mouse (926–3220, Li-cor) and Goat-anti-rabbit (926–68071, Li-Cor).

Antibody used for immunoprecipitation: Myc-tag [9B11 (#2276) or 71D10 (#2278), Cell Signaling Technology], Mouse (G3A1) mAb IgG1 Isotype Control (#5415, Cell Signaling Technology).

Antibodies used for immunofluorescence: panTEAD [D3F7L (#13295), Cell Signaling Technologies], RIF1 (NBP2-26219, Novus biologicals), pS139 H2A.X (05–636, Millipore-Sigma).

### siRNAs and shRNAs

siRNAs for TEAD-1 (L-012603–00), TEAD-2 (L-012611–01), TEAD3 (L-012604), TEAD4 (L-019570–00), DNA ligase 4 (LIG4) (L-004254–00), tumor suppressor P53-binding protein 1 (53BP1) (L-003548–00), and BRCA2 (L-003462–00) are from Horizon Discovery. Nontargeting control siRNA is from Qiagen (1,027,310).

MDA-MB-231 shTEAD_1 target sequences:

TEAD1: 5′-GCTCAAACACTTACCAGAGAA-3′

TEAD2: 5′-ATGACCTGTGAGATCACAAAG-3′

TEAD3: 5′-CCTGGAGTATTCAGCCTTCAT-3′

TEAD4: 5′-GAGACAGAGTATGCTCGCTAT-3′

MDA-MD-231 shTEAD_2 target sequences are the same as the target sequences for shTEAD_1 except for TEAD2 sequence: 5′-GCCTGAGCGATACATGATGAA-3’.

Detroit 562 shRNA target sequences for TEAD are as follows:

shTEAD1/3/4 #1 sequence 5′-ATGATCAACTTCATCCACAAT-3′

shTEAD1/3/4 #2 sequence 5′-GATCAACTTCATCCACAAGCT-3′

These sequences were cloned into PiggyBac (pBind, Genentech), similar to as described in (24).

### Cellular Fractionation, Western Blotting, and Immunoprecipitation

Cellular fractionation between the cytoplasm, nucleus and chromatin fraction was performed using a modified protocol established by Mendez *et al.* (25) using fresh cell pellets resuspended in buffer A [10 mM Hepes, 10 mM KCl, 1.5 mM MgCl_2_, 340 mM sucrose, 10% glycerol, 1 mM dithiothreitol (DTT), 0.1% Triton-X, and 1× Halt Protease and Phosphatase Inhibitor Cocktail (Thermo Fisher Scientific)]. The lysates were incubated on ice for 5 min. Then, the nuclear fraction was isolated by centrifugation (1300*g*) for 4 min at 4 °C. The supernatant (cytoplasm) was collected, and the nuclear pellet was washed several times with buffer A. The nuclei were lysed using buffer B (3 mM EDTA, 0.2 M EGTA, 1 mM DTT, 1× Halt Protease and Phosphatase Inhibitor Cocktail). The supernatant (nucleoplasm) was collected following centrifugation (1700*g*) for 4 min at 4 °C. Then, the cell pellet was washed several times with buffer B. The cell pellet (chromatin) was lysed with RIPA buffer (Thermo Fisher Scientific) according to manufacturer’s instructions, supplemented with 2 mM MgCl_2_, universal nuclease (Thermo Fisher Scientific), and 1× Halt Protease and Phosphatase Inhibitor Cocktail (Thermo Fisher Scientific) for 20 min on ice. For Western blotting, proteins of interest were lysed using RIPA buffer according to manufacturer’s instructions supplemented with 2 mM MgCl_2_, universal nuclease, and 1× Halt Protease and Phosphatase Inhibitor Cocktail for 20 min on ice. Protein concentration was measured by Bradford reagent (5000205, Bio-Rad) using SpectraMax M5 (Molecular devices) at 595 nm. Cell lysates were run on a 4 to 12% Bis-Tris Plus protein gel (Thermo Fisher Scientific) and subsequently transferred onto a nitrocellulose membrane using Transblot Turbo transfer system (Bio-Rad). The membrane was blocked and incubated with primary antibodies in 5% milk for 24 h at 4 °C. After several washes with TBST, membranes were incubated with secondary antibodies: Goat anti-mouse (926–3220, Li-cor) and Goat-anti-rabbit (926–68071, Li-Cor). Images were acquired using the Odyssey Clx (Li-Cor) or by SuperSignal West Pico ECL (1,856,136, Thermo Fisher Scientific)

For immunoprecipitation experiments, expression was induced by doxycycline treatment of MYC/FLAG WT or NLS mutant TEAD3 HEK293 or Detroit 562 cells for 48 h. Cells were harvested, washed with ice-cold PBS, and lysed using 20 mM Tris-HCl pH 7.4, 150 mM NaCl, 0.5% NP-40, 2 mM MgCl_2_, universal nuclease (Thermo Fisher Scientific), and 1× Halt Protease and Phosphatase Inhibitor Cocktail (Thermo Fisher Scientific) for 20 min on ice. 20 mg/ml of whole cell lysates were clarified by centrifugation at 4000*g* 30 min at 4 °C. Immunoprecipitation was carried out on cell lysates using Myc-tag antibody with protein A and G Dynabeads magnetic beads (Thermo Fisher Scientific) according to manufacturer’s instructions, and Mouse IgG1 Isotype Control was used as control to preclear the protein lysates and used for IP. Immunoprecipitated proteins were eluted from the magnetic beads at 95 °C for 10 min in 2× NuPAGE LDS Sample Buffer (Thermo Fisher Scientific) without any reducing agent.

### In-Gel Reduction/Alkylation and Tryptic Digestion

In preparation for mass spectrometry analysis, samples were separated on 4 to 12% Bis-Tris gel (NW04120, Thermo Fisher Scientific) under nonreduced conditions to separate any antibody species from where TEAD proteins run ∼55 kDa. Proteins were stained with SimplyBlue stain (Invitrogen) and de-stained in water. The gel was excised from top to bottom into 14 bands per lane. Gel pieces were further de-stained in 50 mM ammonium bicarbonate (NH_4_HCO_3_)/30% acetonitrile (ACN) and dehydrated in 100% ACN. In-gel reduction was performed in 50 mM NH_4_HCO_3_ containing 50 mM of DTT at 37 °C for 1 h, followed by a quick wash with 50 mM NH_4_HCO_3_ buffer. Alkylation was done using 50 mM iodoacetamide (IAA) at room temperature for 20 min in the dark. Excess amount of IAA was removed by washing the gel pieces with 50 mM NH_4_HCO_3_/30% ACN followed by dehydration in 100% ACN. In-gel tryptic digestion was performed by hydrating the gel pieces in 10 ng/μl trypsin solution in 25 mM NH_4_HCO_3_ and chilled on ice for 1 h. Excess trypsin solution was removed, and digestion was performed overnight in 25 mM NH_4_HCO_3_ at 37 °C. Peptides were extracted with 0.1% trifluoroacetic acid in ACN. Peptides were dried to completion and re-suspended in 2% ACN/0.1% formic acid (FA)/water.

### LC-MS/MS Analysis

Samples were reconstituted in solvent A (2% ACN/0.1% FA/water) and injected *via* an auto-sampler for separation by reverse phase chromatography on a NanoAcquity UPLC system (Waters). Peptides were loaded onto the Symmetry C_18_ column (1.7 mm BEH-130, 0.1 × 100 mm, Waters) with a flow rate of 1 μl/min. A gradient of 2% solvent B to 25% solvent B (solvent A is 0.1% FA/2% ACN/water and solvent B is 0.1% FA/2% water/ACN) was applied over 35 min with a total analysis time of 60 min. Peptides were eluted directly into an Advance CaptiveSpray ionization source (Michrom BioResources/Bruker) with a spray voltage of 1.3 kV and were analyzed using an LTQ Orbitrap Elite mass spectrometer (Thermo Fisher Scientific). Precursor ions were acquired in the FTMS at 60,000 resolution; MS/MS was performed in the LTQ using resonance excitation, with the instrument operated in data-dependent mode, whereby the 15 most abundant ions were selected for fragmentation in each duty cycle.

### Mass Spectrometry Data Analysis and Statistical Analysis

For peptide identification, MS/MS spectra were searched using the search algorithm Mascot version 2.4.1 (Matrix Sciences) against the concatenated target-decoy database comprised of UniProt human protein sequences (UniProt, version 2016_06 that includes 42,279 Swissprot sequences of canonical and protein isoforms and 134,256 unreviewed TREMBL sequences), one custom NLS mutant TEAD sequence, known contaminants, and the reversed versions of each sequence. A 50 ppm precursor ion mass tolerance and 0.8 Da fragment ion tolerant were selected with tryptic specificity with up to three miscleavages. Variable modifications were permitted for methionine oxidation (+15.9949 Da), IAA adduct for cysteine residues (+57.0215 Da), phosphorylation on serine, threonine, and tyrosine (+79.9663 Da), and ubiquitination on lysine residues (+114.0429 Da). Peptide assignments were first filtered to a 1% false discovery rate at the peptide level and subsequently at 2% false discovery rate at protein level. We used SAINTexpress (Significance Analysis of INTeractome), which is a statistical method for probabilistically scoring protein-protein interaction data from AP-MS experiment (SAINTExpress-spc v.3.6.1) (Teo *et al*., 2014). We compared spectral counts for each bait IP sample (WT-TEAD3 or NLS-mutant TEAD3) and to control IP samples and to assign confidence scores to observed protein-protein interactions. Protein spectral counts for each sample were calculated at the sum from the peptide spectral counts (all modified and shared peptides) for one protein in a given sample, across all fractions from the GeLC-MS experiment. No filtering for protein spectral counts was performed. A single peptide is also used for protein quantification. The default setting for SAINTexpress was used (low Mod = 0, minFold = 1, normalization = 1). We did not compress control or bait samples. Interactions with average sum peptide spectral matches >10, a SAINT score >0.99, and a Bayesian False Discovery Rate < 0.001 were marked as significant. Significant interactions for the WT and NLS mutant TEAD3 in each cell line were submitted for ‘Compute overlaps’ tool against the Hallmark gene sets from MsigDB (v2022.1.Hs updated August 2022) (26) using the Broad institute’s online tool (26, 27). Hallmark gene sets with a q-value <0.05 were marked as significant.

### Immunofluorescence

Cells were either cultured on 1.5 glass coverslips (cat. 64–0714, Warner instruments) or 96-well plates, fixed with 4% paraformaldehyde for 10 min at room temperature, permeabilized with Triton buffer (0.5% Triton X-100, 20 mM Hepes, pH 7.9, 50 mM NaCl, 3 mM MgCl_2_, and 300 mM sucrose). Then, cells were washed with PBS (2×) followed by blocking using 2% BSA for 1 h at room temperature. Primary antibodies were incubated in 0.1% Tween-20 in 2% BSA overnight, followed by three washes with 0.1% Tween-20/PBS. Secondary antibodies were incubated for 1 h in 0.1% Tween-20 in 2% BSA. Cells were counterstained with DAPI for the last 10 min of secondary incubation, followed by three quick washes with 0.1% Tween-20/PBS. Coverslips were mounted onto slides using VectaMount (H-5000, Vector Labs.)

### TEAD Transcriptional Reporter Assay

Detroit 562 and MDA-MB-231 were transduced with a lentiviral vector expressing secreted NanoLuc luciferase under the control of multimerized 8xTEAD consensus element (5′-CACATTCCA-3′). Within the same construct, Firefly Luciferase is constitutively expressed from a PGK Promoter and serves as an internal control (schematic in supplemental Fig. S2*A*). 10,000 cells were seeded per well in a 96-well plate and incubated for 24 h. Cells were then treated with 500 nM dasatinib, exposed to 6 Gy of γIR, or left untreated. At the indicated time points posttreatment (4, 6, 24, 30, and 48 h), TEAD reporter activity was assayed by performing Nano-Glo Dual-Luciferase Reporter Assay System (Promega N1610) following the manufacturer’s instructions. Plates were read on an Envision multimode microplate reader. TEAD reporter activity was calculated by normalizing the NanoLuc luminescence signal to the Firefly Luciferase signal, where the Luciferase signal represents the overall cell viability.

### DNA Repair Reporter Assay

To test homologous recombination (HR) efficiency, we used pDR-GFP in U-2 OS cells, originally developed in the Jasin lab (28). siRNAs for various targets were transfected using Lipofectamine RNAiMAX (Thermo Fisher Scientific) according to the manufacturer’s instructions. After 24 h, transfection of the plasmid encoding I-SceΙ was performed with Lipofectamine 2000 (Thermo Fisher Scientific) according to the manufacturer’s instructions. Cells were allowed to repair the DSBs for at least 48 h before GFP analysis by fluorescence-activated cell sorting using Attune N × T Flow Cytometer. Recombination efficiencies were calculated as % GFP positive cells relative to siRNA control: (% GFP positive siRNA X)/(% GFP positive siRNA control) ∗100.

To test nonhomologous end joining repair (NHEJ) efficiency, we used iHN HeLa-Tet cell line (DR5001-iHNHeLa-Tet, TopoGEN) according to manufacturer’s instructions. After siRNA transfection as above, cells were treated with 2 μg/ml doxycycline to induce I-Sce1 mediated DSBs for 24 h. Cells were then allowed to repair DSBs for at least 48 h before GFP analysis by fluorescence-activated cell sorting using BDCelesta analyzer (BD Biosciences). The % repair efficiency is the fraction of GFP positive cells in siRNA treated samples normalized to the fraction of GFP positive cells in control siRNA treated cells. Data were quantified using FlowJo software. Recombination efficiencies were calculated as % GFP positive cells relative to siRNA control: (% GFP positive siRNA X)/(% GFP positive siRNA control) ∗100.

### Comet Assay

The Comet assay was performed using OxiSelect comet assay kit (STA-351, Cell Biolabs, Inc.) following manufacturer’s instructions. MDA-MB-231 cells expressing nontargeting shRNA control (shNTC), shTEAD_1 or shTEAD_2 were treated with DMSO or bleomycin for 2 h. Cells were allowed to recover in fresh media for 24 h. Then, cells were gently trypsinized and pelleted by centrifugation. To form a base layer, 75 μl of molten LMAgarose was pipetted onto the comet slide and allowed to solidify at 4 °C for 15 min. Cells were resuspended at 1 × 10^5^ cells/ml in cold PBS, followed by combining cells with molten LMAgarose at a ratio of 1:10 (v/v), and quickly pipetting 75 μl onto a comet slide, without disrupting the base gel layer. The slide was placed at 4 °C in the dark for 15 min, then immersed in precooled lysis buffer and kept at 4 °C in the dark for 45 min. The comet slide was immersed in prechilled alkaline solution for 30 min. Then, the comet slide was transferred to a horizontal electrophoresis apparatus to perform alkaline electrophoresis at 20 V for 25 min. Samples were then rinsed three times with prechilled PBS for 5 min. Samples were dried at room temperature in the dark, then stained with 100 μl of diluted SYBR Green I Nucleic Acid Gel Stain (S7567, Thermo Fisher Scientific Scientific) for 30 min. Images were captured by a confocal microscope (SP5, Leica microsystems) from four different fields and data were analyzed by CometScore.

### Survival Assay

Fifty thousand cells were initially seeded in 6-well dishes before being exposed to 6 Gy γIR or being treated with the indicated concentrations of bleomycin or etoposide. At 24 h post γIR exposure or treatment, cells were incubated with fresh media and incubated for 5 to 6 days. Thereafter, cells were fixed with 10% glacial acetic acid and 10% methanol for 10 min and stained with 0.5% crystal violet. After several rinses with ddH_2_O and allowing the plates to dry, the crystal violet was solubilized with 0.01% SDS in methanol. The absorbance was measured using SpectraMax M5 (Molecular devices) at 595 nm.

### Cell Cycle Analysis

HeLa and U-2 OS cells were transfected with siControl or siTEAD using Lipofectamine RNAiMAX (Thermo Fisher Scientific) according to the manufacturer’s instructions. After 72 h, cells were treated with 10 μM EdU for 15 min at 37 °C, fixed with 4% PFA at room temperature, and permeabilized and blocked in PBS containing 10% FBS, 1% BSA, 0.1% TX-100, and 0.01% NaN_3_ for 1 h at room temperature. Click reaction was then performed following manufacturer's instructions (Thermo Fisher plus EdU cell proliferation kit). Cells were washed with PBS, re-blocked for 30 min, and treated with Hoechst 33,342 1:10,000 in PBS for 10 min. After washing out Hoechst, cells were imaged on OperaPhenix (PerkinElmer). Images were segmented and quantified following the protocol outlined in (29).

## Results

### Nuclear Localization of TEADs Promotes Interaction With Proteins Involved in DNA Repair

**Fig. 1:**
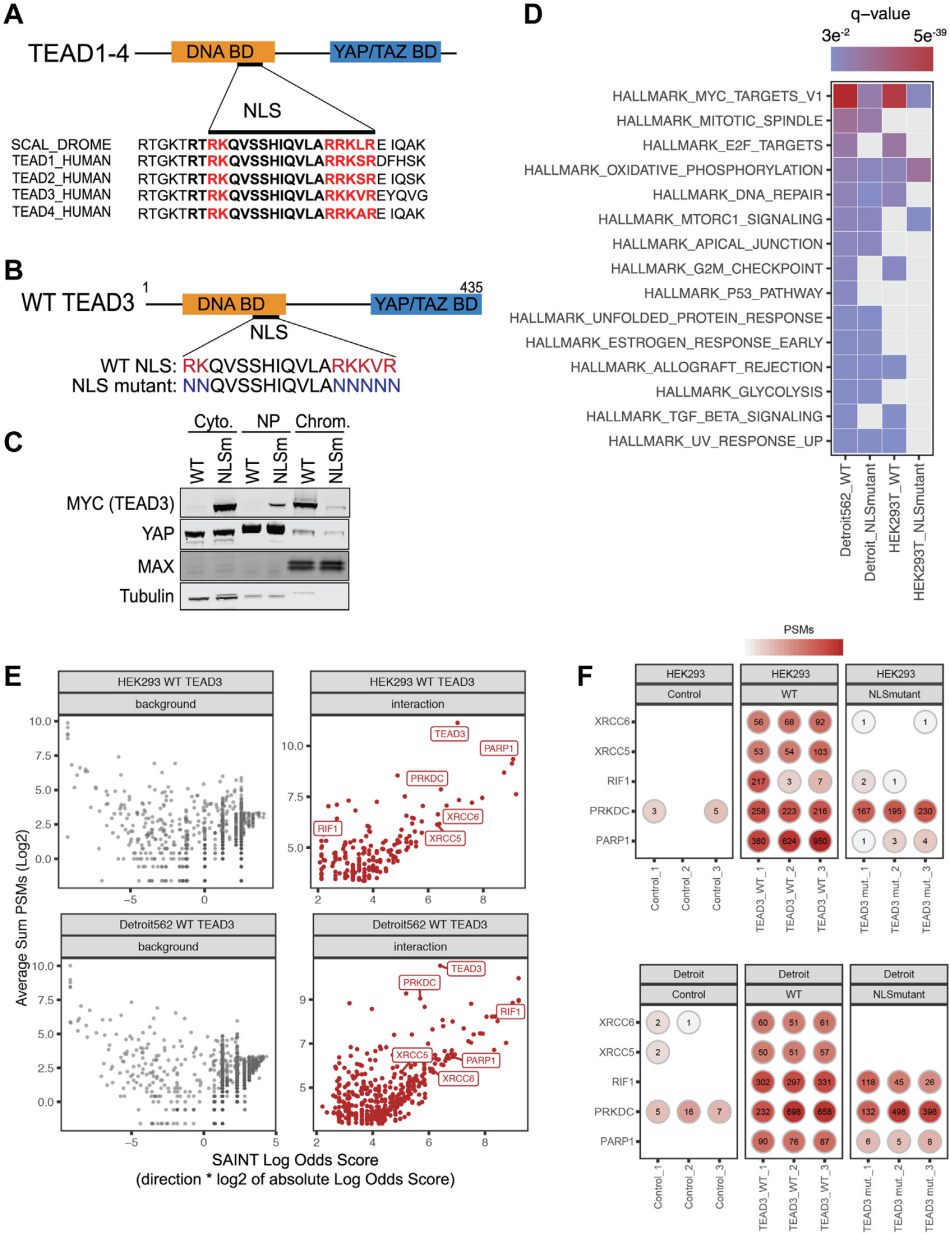
**Proteomic analysis of TEAD3 identifies DNA-damage-associated proteins as TEAD-interacting partners**. *A*, human TEAD proteins 1 to 4 contain a conserved nuclear localization signal (NLS). Protein schematic and sequence alignment of the NLS of *D. melanogaster* protein Scalloped to human TEAD proteins. *B*, schematic of the TEAD3 NLS mutant generated by mutation of seven NLS residues to asparagine. *C*, subcellular fractionation of MYC-WT and MYC-NLS mutant TEAD3. Cyto. = cytoplasm, NP = nucleoplasm, Chrom. = Chromatin. This is a representative image from three or more replicates. *D*, heatmap showing the enriched Hallmark gene sets from MSigDB (FDR q-value <0.05) for WT and NLS mutant TEAD3 interactions in both cell lines. The enriched hallmark gene sets were sorted by the q-value of Detroit WT, and the top 15 gene sets are shown in the heatmap. *E*, scatter plot representing the abundance (Average Sum PSMs, log2 scale) and Specificity (SAINT Log Odds Score, direction ∗ log2 scale of absolute Log Odds Score) for all identified proteins by AP-MS in the TEAD3 WT compared to negative control. Interactions with a SAINT score >0.99, average sum PSMs >10, a SAINT score >0.99, and a Bayesian false discovery rate (BFDR) < 0.001 were classified as significant interactions (*red*), while the others were marked as background (*light gray*). *F*, dotplot representing the PSMs for selected hits, marked in (*E*), per cell line and IP for each replicate. AP-MS, affinity purification mass spectrometry; PRKDC, DNA-dependent protein kinase, catalytic subunit; PSM, peptide spectral match; PARP1, poly(ADP-ribose) polymerase 1; RIF1, Rap1-interacting factor 1; TEADs, TEA domain family members 1 to 4; XRCC6, X-ray repair cross-complementing protein 6; XRCC5, X-ray repair cross-complementing protein 5; YAP, Yes-associated protein.

The identification of an NLS within TEADs and Scalloped in *Drosophila melanogaster* led us to investigate whether we could identify novel nuclear modulators of TEADs in humans (3, 30). Alignment of the four human TEAD proteins and *D. melanogaster* Scalloped demonstrates a conserved NLS region within the DNA binding domain (3) (Fig. 1*A*). We designed a mutant of TEAD3 to disrupt the NLS by mutating seven residues to asparagine (Fig. 1*B*). Next, we generated doxycycline-inducible stable cell lines expressing either MYC-tagged WT-TEAD3 or MYC-tagged NLS-mutant TEAD3 in HEK293 cells and Detroit 562 cells (a YAP-amplified cell line). Using subcellular fractionation, we found that nuclear localization was indeed disrupted by our mutant. WT TEAD3 was enriched in the chromatin fraction, while the NLS-mutant TEAD3 was predominantly enriched in the cytoplasm (Fig. 1*C*).

We next performed semiquantitative mass spectrometry analysis of immunopurified MYC-tagged WT-TEAD3 or MYC-tagged NLS-mutant TEAD3 to identify binding partners of TEAD3. We performed these experiments in two different cell lines, HEK293 and the YAP-amplified cell line Detroit 562. In total, we identified 2749 proteins in HEK293 cells and 3015 proteins in Detroit 562 cells (supplemental Tables S1 and S2). Scatter plots of the spectral count data show similar signal and variability between the replicates, and PLN-PCA plot analysis (31, 32) shows some variability (supplemental Fig. S1, *A* and *B* for HEK293 cells and supplemental Fig. S1, *C* and *D* for Detroit 562 cells; supplemental Table S2). One replicate for NLS mutant bait and one replicate for WT are separated from other biological replicates in the same group in both cell types, but overall, the replicates cluster similarly (supplemental Fig. S1, *B* and *D*). To identify significant interactions, we next performed SAINT analysis using SAINTexpress (33) (supplemental Table S3). For HEK293 cells, SAINT analysis comparisons to the negative control (Isotype IgG IP) identified 191 significant interacting proteins for WT TEAD3 and 63 significant interacting proteins for NLS mutant TEAD3. For Detroit 562 cells, SAINT analysis comparisons to the negative control identified 429 and 242 significant interacting proteins for WT TEAD3 and NLS mutant TEAD respectively.

To evaluate how the WT TEAD3 may differ in function compared to the NLS mutant, we next performed functional gene enrichment analysis on the identified significant interacting proteins. Using the Hallmark gene sets from the Molecular Signature Database, MSigDB (26), we found that hallmarks of E2F, G/M checkpoint, and DNA repair pathways were differentially represented between WT and NLS-mutant TEAD3 (Fig. 1*D* and supplemental Table S4). We identified several DNA repair proteins as interacting partners enriched with WT-TEAD3 compared to negative control (Fig. 1*E*). Among the most abundant proteins that were identified in both HEK293 and Detroit 562 cell lines were factors involved in repairing double-stranded DNA breaks including poly(ADP-ribose) polymerase 1 (PARP1), RIF1, X-ray repair cross-complementing proteins 5 and 6 (XRCC5 and XRCC6), and DNA-dependent protein kinase, catalytic subunit (PRKDC/DNAPKcs) (Fig. 1*F*).

### Validation of TEAD3 Interaction With DNA Damage Proteins

**Fig. 2:**
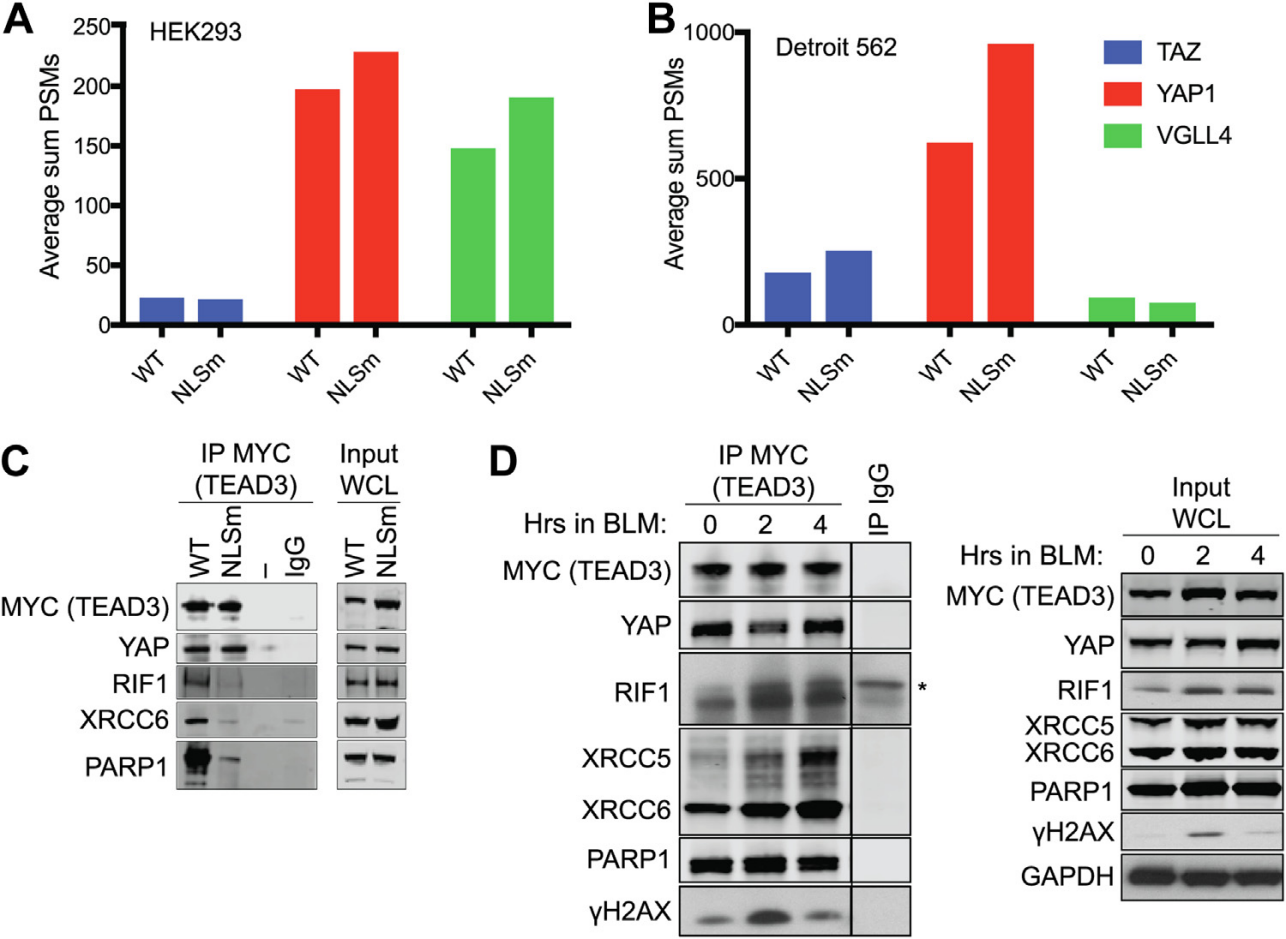
**TEADs interacts with DNA repair proteins**. *A*, bar graph representing the abundance (average sum PSMs) of canonical binding partners, YAP, TAZ, and VGLL4 binding to TEAD equally in HEK293 cells. *B*, as described in (*A*) but for Detroit 562 cells. *C*, co-immunoprecipitation assay comparing MYC-tagged WT TEAD3 and MYC-tagged NLS mutant (NLSm) TEAD3. Figure is representative of three or more replicates. WCL, whole cell lysate. *D*, co-immunoprecipitation of MYC-tagged WT TEAD3 after treatment with 20 μg/ml bleomycin (BLM) for the indicated time points. Figure is representative of three or more replicates. ∗indicates a nonspecific band detected along with RIF1. RIF1, Rap1-interacting factor 1; RIF1, Rap1-interacting factor 1; TEADs, TEA domain family members 1 to 4; TAZ, transcriptional co-activator with PDZ-binding motif; PARP1, poly(ADP-ribose) polymerase 1; XRCC6, X-ray repair cross-complementing protein 6; XRCC5, X-ray repair cross-complementing protein 5; YAP, Yes-associated protein.

Examining the TEAD-associated proteins from our AP-MS data, we found that the TEAD canonical binding partners, YAP, TAZ, and VGLL4, are unchanged by the NLS mutant in both HEK293 cells and Detroit 562 cells (Fig. 2, *A* and *B*), possibly because the NLS mutation occurs near the N terminus and not at the YAP/TAZ binding domain. However, the mass spectrometry data indicate that DNA damage-related proteins do differentially associate with WT and NLS-mutant TEAD3 (Fig. 1, *D*–*F*). To validate this finding, we performed immunoprecipitation followed by Western blot analysis. Consistent with the mass spectrometry results, RIF1, XRCC6, and PARP1 specifically co-immunoprecipitated with WT-TEAD3, while YAP was co-immunoprecipitated with both the WT- and NLS-mutant TEAD3 (Fig. 2*C*). The association of TEAD proteins with DNA repair proteins potentially indicates a role for TEAD in DNA damage repair. To test this hypothesis, we performed co-immunoprecipitation experiments with cell lysates overexpressing WT-TEAD3 treated with or without bleomycin, a radiomimetic drug that induces DSBs. Upon bleomycin treatment, we find increased association of RIF1, XRCC5, XRCC6, and histone H2AX phosphorylated on S139 (γH2AX) with TEAD3, while PARP1 was unaffected (Fig. 2*D*). These results indicate that TEAD proteins may be co-localizing and acting with mediators of DNA damage repair pathways.

### TEADs Are Required for Maintaining Genome Stability

**Fig. 3:**
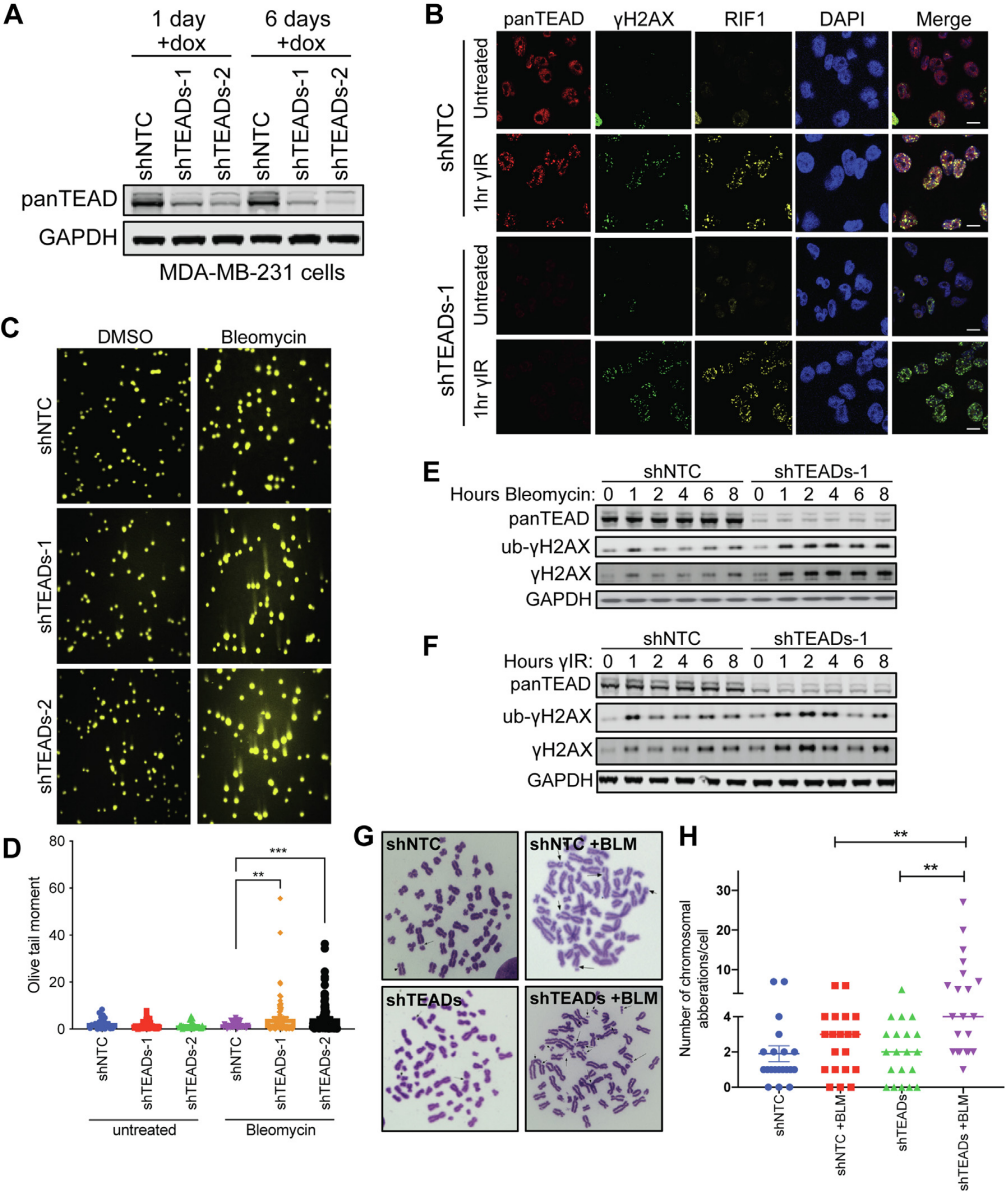
**Depletion of TEAD proteins exacerbates DNA damage and promotes genomic instability.***A*, knockdown of TEAD proteins in MDA-MB-231 using shRNAs targeting all four TEAD paralogs (shTEADs) or a nontargeting control (shNTC). *B*, immunofluorescence of MDA-MB-231 cells containing inducible shNTC or shTEADs-1 treated with or without 6 Gy γIR for 1 h. *C*, comet assay using MDA-MB-231 cells containing inducible shNTC, shTEAD-1, or shTEAD-2. Cells were treated for 2 h with DMSO or 20 μg/ml bleomycin, then allowed to repair for 24 h before assaying. Data are representative of three or more replicates. *D*, quantification of the data represented in (*C*). Three or more replicates were performed for each condition, with four or more fields of view captured for each replicate. The number of cells quantified per condition are as follows: shNTC n = 23, shTEADS-1 n = 42, shTEADS-2 n = 36, shNTC +BLM n = 42, shTEADS-1 +BLM n = 90, shTEADS-2 +BLM n = 99. ∗∗ *p* < 0.01, ∗∗∗ *p* < 0.001 by unpaired two-tailed *t* test with Welch’s correction. *E*, time course analysis of DNA damage markers. MDA-MB-231 cells expressing shNTC or shTEAD-1 were treated with or without 20 μg/ml bleomycin and cells were collected at the indicated time points for analysis by western blotting. *F*, time course analysis of DNA damage markers. MDA-MB-231 cells expressing shNTC or shTEAD-1 were treated with or without 6 Gy γIR, and cells were collected at the indicated time points for analysis by western blotting. *G*, chromosomal spread assay in MDA-MB-231 cells expressing shNTC or shTEAD-1, untreated or treated for 2 h with 20 μg/ml bleomycin, then allowed to repair for 24 h before assaying. *H*, quantification of the data represented in (*G*). Twenty cells were quantified per condition. ∗∗ *p* ≤ 0.01 by unpaired two-tailed *t* test. γIR, γ-irradiation; BLM, bleomycin; Gy, Gray unit; RIF1, Rap1-interacting factor 1; TEADs, TEA domain family members 1 to 4.

Next, we hypothesized that if TEADs participate in DNA repair, then they might also be recruited to DNA damage sites. To address this, we generated cells depleted of TEADs using doxycycline-inducible shRNA knockdowns. Using the breast cancer cell line MDA-MB-231, we compared a set of shRNAs targeting all four TEAD paralogs to a shNTC (Fig. 3*A*). We used these cell lines to test whether TEAD proteins localize with DNA damage-induced nuclear foci.

Using an antibody that detects all four TEAD paralogs and immunofluorescence, we found that TEADs were diffusely expressed in the nucleus of untreated cells (Fig. 3*B*). Surprisingly, TEADs formed nuclear foci after 1 hour of γIR, a property commonly found in DNA repair proteins post-DNA damage (Fig. 3*B*). In addition, we found that TEADs co-localized with DNA damage markers, γH2AX and RIF1, supporting the hypothesis that TEADs play a role in DNA repair (Fig. 3*B*). Notably, depletion of TEADs by shRNA did not affect the localization of γH2AX and RIF1, indicating the TEADs do not modulate these markers of DNA damage (Fig. 3*B*). Additionally, we found that YAP or TAZ did not form DNA damage induced nuclear foci but rather are localized diffusely in the nucleus in MDA-MB-231 cells (supplemental Fig. S2*A*). Furthermore, we tested whether the formation of DNA damage foci is independent of YAP and TAZ. Assaying SKNF1, a cell line with no detectable YAP or TAZ expression (supplemental Fig. S2*B*), we observed that TEADs still formed nuclear foci that co-localized with γH2AX and RIF1 after γIR (supplemental Fig. S2*C*). Given this observation that TEADs localization to DNA damage foci occurs even in the absence of YAP and TAZ, we next asked whether TEAD-mediated transcription was affected after DNA damage by using a 6xGT-TEAD responsive luciferase reporter in MDA-MB-231 and Detroit 562 cells (supplemental Fig. S2*D*). In both cell lines, treatment with dasatinib (34), a tool compound that induces the phosphorylation and inhibits the activity of YAP/TAZ, dramatically suppressed luciferase signal over time, while treatment with γIR had little if any effect on TEAD-mediated transcription (supplemental Fig. S2, *E* and *F*). Altogether, these data provide observation of a potential role for TEADs in DNA repair and indicate that DNA damage does not majorly impair the transcriptional activity of a YAP/TAZ–TEAD reporter.

Next, using a comet assay (35), a method for measuring the magnitude of DNA breaks, we tested if shRNA-mediated depletion of TEADs impacted the extent of DNA damage. We found that depletion of TEADs combined with treatment with bleomycin significantly increased DNA breaks, although depletion of TEADs alone was not significantly different from the DMSO control (Fig. 3, *C* and *D*). Moreover, the levels of γH2AX and monoubiquitinated γH2AX, which is a marker of nonapoptotic DNA DSBs (36), persisted after DNA damage in cells depleted of TEADs (Fig. 3, *E* and *F*). Next, we tested chromosomal instability in TEADs-depleted cells by performing metaphase chromosome spreads. While depletion of TEADs alone did not increase chromosomal aberrations, treatment with bleomycin in combination with depletion of TEADs significantly increased chromosomal aberrations including breakages, nicks, and translocations (Fig. 3, *G*–*H*). Altogether, these data suggest that TEADs are required to efficiently prevent the accumulation of DNA damage and to maintain genomic stability.

### Depletion of TEADs by RNAi Sensitizes Cells to DNA Damage

**Fig. 4:**
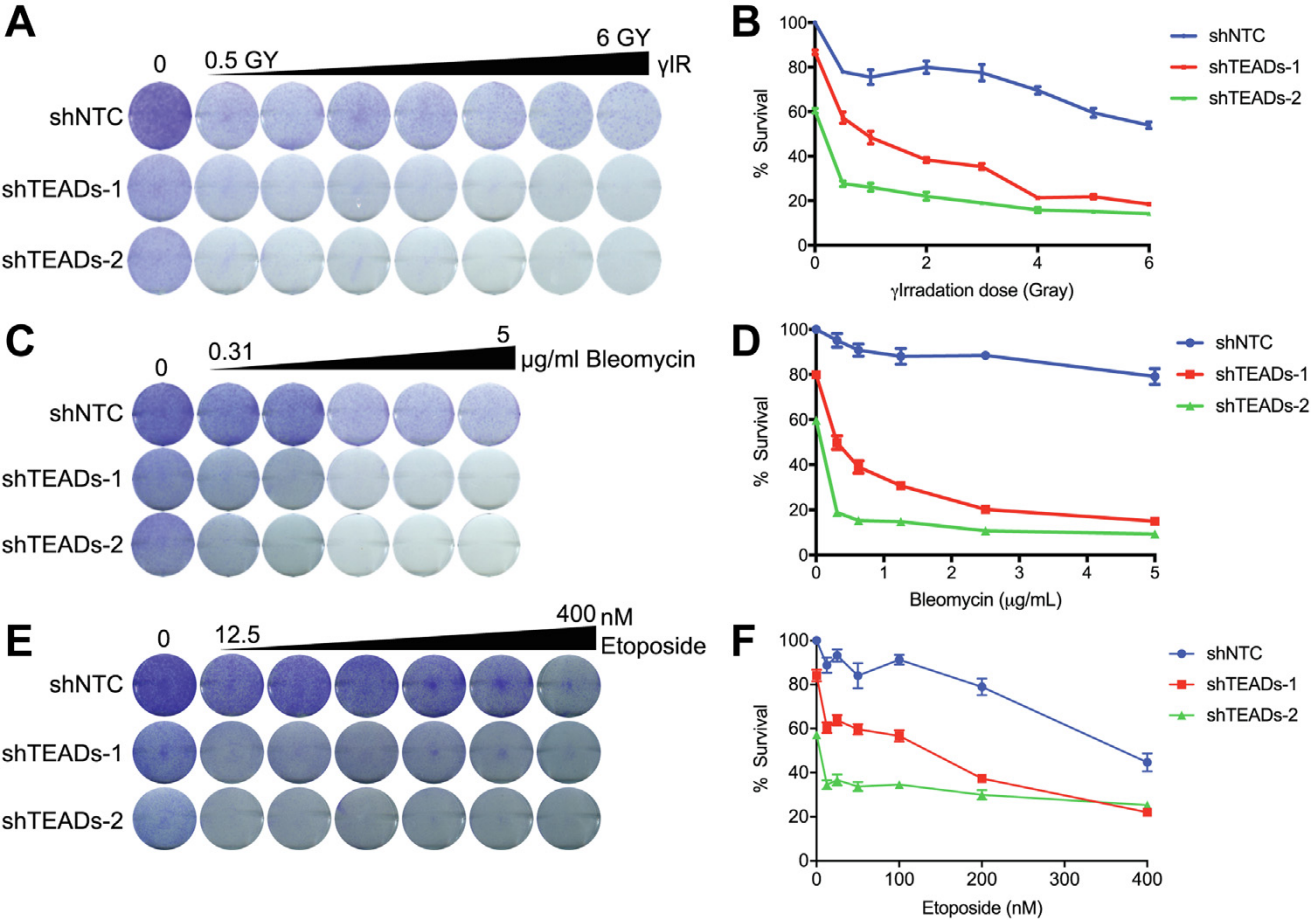
**Depletion of TEADs decreases cellular resistance to DNA damage**. *A*, MDA-MB-231 cells expressing shNTC, shTEADs-1, or shTEADs-2 were treated with increasing doses of γIR for 24 h. Cells were then exchanged into fresh media and incubated for 5 to 6 days before fixation and staining with crystal violet to quantify cell growth. *B*, quantification of the data represented in (*A*). *C*, MDA-MB-231 cells expressing shNTC, shTEADs-1, or shTEADs-2 were treated with increasing concentrations of bleomycin for 24 h. Cells were then exchanged into fresh media and incubated for 5 to 6 days before fixation and staining with crystal violet. *D*, quantification of the data represented in (*C*). *E*, MDA-MB-231 cells expressing shNTC, shTEADs-1, or shTEADs-2 were treated with increasing concentrations of etoposide for 24 h. Cells were then exchanged into fresh media and incubated for 5 to 6 days before fixation and staining with crystal violet. *F*, quantification of the data represented in (*E*). shNTC, nontargeting shRNA control; TEADs, TEA domain family members 1 to 4.

To test the functional significance of TEADs in DNA repair, we examined cell growth sensitivity to DNA damage in TEADs-depleted cells. We used MDA-MB-231 cells and quantified cell growth with crystal violet staining. Depletion of TEADs in MDA-MB-231 moderately slowed cell growth, and treatment with the DNA damaging agents γIR, bleomycin, and etoposide further sensitized these cells (Fig. 4, *A*–*F*). Similar results were observed upon depletion of TEADs in Detroit 562 (supplemental Fig. S3, *A–C*). These results suggest that TEADs may influence cellular responses to DSBs.

### Depletion of TEADs Influences Cellular Repair Response to Double-Stranded Breaks

Pathways of DNA damage repair safeguard the genome from endogenous and exogenous sources of DNA damage, including DSBs. If left unrepaired, these lesions can compromise genome integrity and lead to cancer (37). Two cellular pathways resolve these potentially detrimental aberrations to DNA: NHEJ and HR (37, 38). In NHEJ, DSBs are repaired very quickly by joining two DNA ends with very little sequence homology, an error-prone repair pathway. In HR, DSBs are repaired through a multistep process that involves extensive DNA resection around the DSB and sequence homology from sister chromatids. Notably, DNA repair by HR can only occur during S-phase of the cell cycle when the sister chromatids are open and available as templates for efficient repair.

**Fig. 5:**
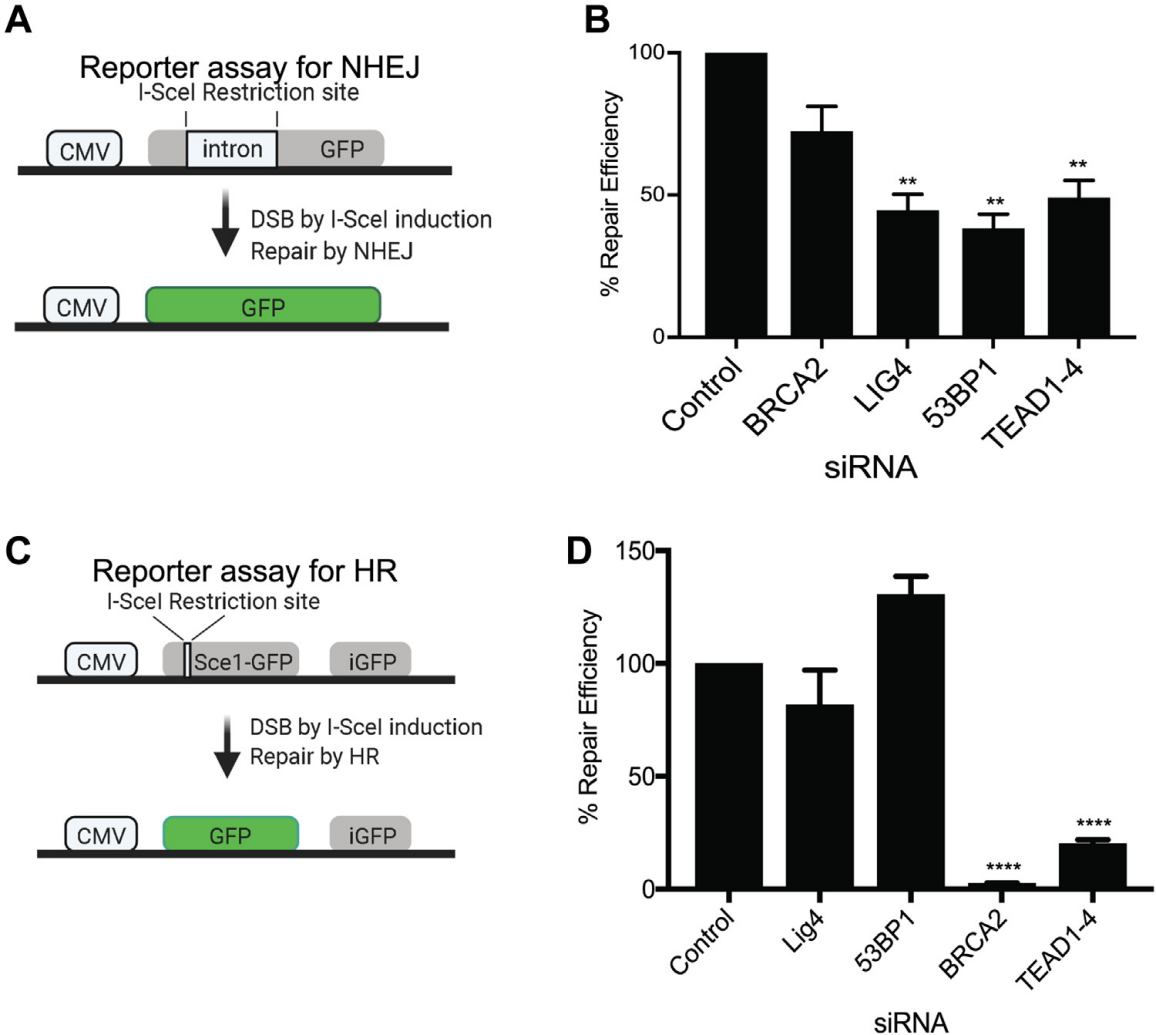
**Depletion of TEADs by RNAi decreases NHEJ and HR DNA repair in response to double-stranded break**s. *A*, schematic of the NHEJ GFP-reporter assay in HeLa cells. *B*, depletion of TEAD1-4 by siRNA significantly decreased NHEJ repair efficiency. Positive controls for NHEJ are siLIG4 and si53BP1. Three biological replicates were performed, and at least 10,000 cells were analyzed per condition. Statistical analysis was performed using GraphPad Prism software, ∗∗*p* < 0.01. *C*, schematic of the direct-repeat (DR) GFP-reporter assay for HR in U-2 OS cells. *D*, depletion of TEAD1-4 by siRNA significantly decreased recombination repair efficiency. The positive control for HR is siBRCA2. Three independent experiments and three technical replicates in each experiment were performed. In all cases, 10,000 cells/well were plated and cells were analyzed using an Attune N × T Flow cytometer after 48 h. Statistical analysis was performed using GraphPad Prism software, ∗∗∗∗*p* < 0.0001. HR, homologous recombination; NHEJ, nonhomologous end joining; TEADs, TEA domain family members 1 to 4.

**Fig. 6:**
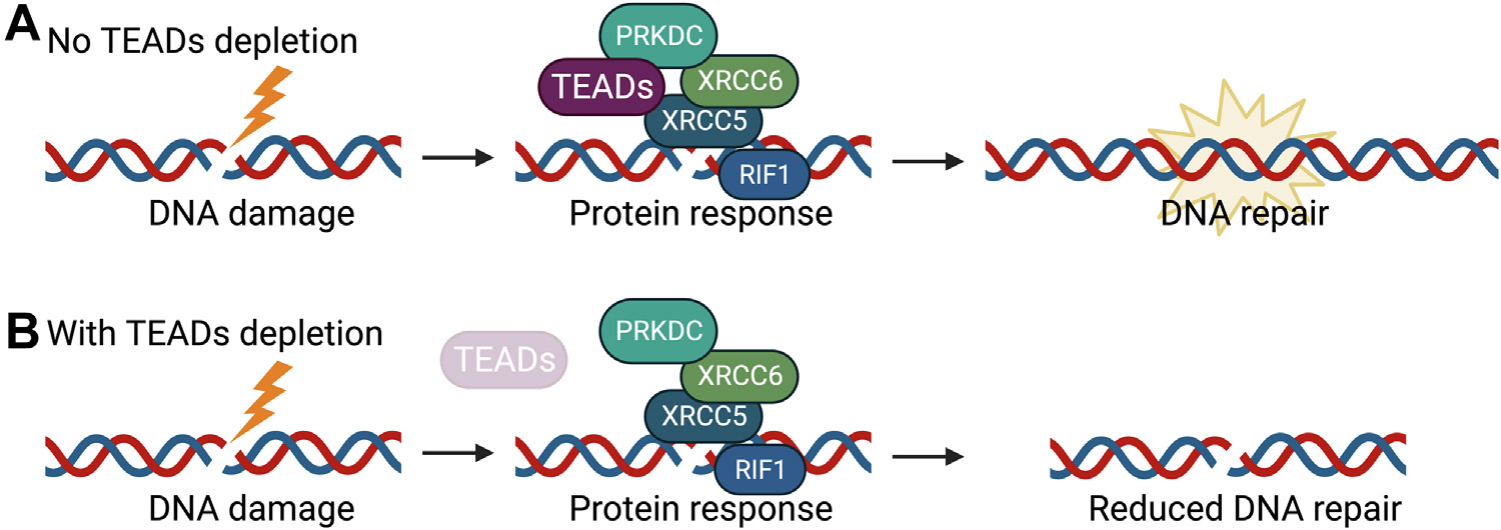
**Schematic of the connection between TEADs and DNA damage repair**. *A*, the interaction of TEAD with canonical DNA repair protein machinery facilitates repair of double stranded breaks, protecting cells from deleterious damage. *B*, depletion of TEADs results in decreased efficiency of double stranded break DNA repair. PRKDC, DNA-dependent protein kinase, catalytic subunit; RIF1, Rap1-interacting factor 1; TEADs, TEA domain family members 1 to 4; XRCC6, X-ray repair cross-complementing protein 6; XRCC5, X-ray repair cross-complementing protein 5.

Since our data indicate that TEADs interact with several proteins involved in sensing and repairing DSBs, we next examined whether TEADs directly regulate NHEJ and HR. To assess NHEJ, we used a commercially available GFP-based reporter cell line (TopoGen HeLa NHEJ Reporter Cell Line), and to assess HR, we used a GFP-based cell line reporter in U-2 OS cells (39). These assays test repair efficiency by measuring the relative levels of successful repair of a GFP reporter cassette in cells (Fig. 5, *A* and *C*). As controls for NHEJ, we used siRNA knockdown of two key NHEJ regulators, LIG4, and 53BP1. As a control for HR, we used siRNA depletion of the HR regulator BRCA2. Indeed, depletion of the key NHEJ regulators LIG4 and 53BP1 decreased NHEJ efficiency (Fig. 5*B*), and depletion of HR regulator BRCA2 decreased NHEJ efficiency (Fig. 5*D*). Interestingly, depletion of TEADs significantly decreased both NHEJ repair efficiency and HR repair efficiency, suggesting that TEADs are involved in both mechanisms of DNA repair (Fig. 5, *B* and *D*). Since HR preferentially occurs in S-phase of the cell cycle, we tested whether the HR inhibition is simply a consequence of differing cell cycle progression in cells depleted of TEADs. Interestingly, depletion of TEADs did not change the cell cycle profile in the HeLa NHEJ reporter cell line (supplemental Fig. S4, *A* and *B*) or in the U-2 OS HR reporter cell line (supplemental Fig. S4, *C* and *D*). These data suggest that TEADs may influence efficient DNA repair by both NHEJ and HR. Therefore, perhaps the association of TEADs with the proteins, RIF1, XRCC5, XRCC6, PRKDC, and PARP1, identified by AP-MS influence the processes that maintain genomic integrity (Fig. 6).

## Discussion

DDR is a network of processes that safeguard genomes from deleterious endogenous and exogenous DNA damage. If left unrepaired, DNA damage can produce irreversible mutations that may lead to various deleterious effects, including cancer. Here, we identified binding partners for TEADs by performing semiquantitative mass spectrometry analysis using cell lines overexpressing either WT-TEAD3 or NLS-mutant TEAD3. We found that PARP1, RIF1, and XRCC5 and XRCC6 were enriched in the WT-TEAD3 samples but were absent or significantly decreased in the NLS-mutant TEAD3 samples. In contrast, the canonical binding partners of TEADs, YAP, TAZ, and VGLL4, displayed no change in interaction between mutant and wildtype conditions. Because the NLS region of TEADs is highly conserved, we anticipate that the results we observe with TEAD3 likely apply to all four TEAD paralogs, although future experimental testing is required to validate such interactions with TEAD1, TEAD2, and TEAD4. Notably, previous studies have identified XRCC5 and XRCC6 as interacting partners for TEAD2 and TEAD4 (15).

To evaluate the role of TEADs associating with these DNA damage markers, we used RNAi-based depletion systems targeting all four TEADs as well as agents that induce DNA damage. Upon the introduction of DNA damage in cells, we observed that TEADs are recruited to sites of DSBs, co-localizing with DNA damage markers γH2AX and RIF1. We also observed that interaction of TEADs with XRCC5 and XRCC6 is increased after DNA damage. Additionally, depletion of TEADs by RNAi reduces the efficient maintenance of genome stability, sensitizes cells to agents that cause DSBs, and reduces DNA repair in both HR and NHEJ GFP reporter assays. These cellular findings indicate that TEADs play a role in DDR; however, the RNAi-centered approach for TEADs depletion we used in this study is an important limitation due to potential off-target effects that are inherent to RNAi. Follow-up experiments that disrupt TEADs in other ways, such as with CRISPR approaches or with TEAD inhibitors, would provide valuable insights in evaluating the functional significance of TEADs in maintaining genomic stability.

Although TEADs function as transcription factors, perhaps they have an additional role in regulating DNA repair after DNA damage to restore cellular homeostasis. Several studies have shown that other transcription factors play an important role in DDR; for instance, several transcription factors localize to DNA damage sites, some of which are PARP1-dependent (40). However, the exact physiological role of these transcription factors at the DNA damage sites remains unknown. Several independent studies have begun to unravel the role of transcription factors in DNA repair. Most notably, E2F transcription factor 2 functions to regulate genes involved in proliferation and apoptosis and has been implicated to directly participate in DNA repair by forming DNA damage–induced foci mediated by ataxia telangiectasia mutated (ATM) and ATM- and Rad3-related after ultraviolet-induced DNA damage (41, 42). ATF2 is another transcription factor belonging to the AP1 family of stress responsive transcriptional activators and has been shown to localize at sites of DSBs by ATM-induced phosphorylation (43). POU domain transcription factor, BRN2, has been shown to bind to and be recruited by PARP1 along with XRCC5 and XRCC6 to sites of ultraviolet B-induced DNA damage to suppress apoptosis (44). These studies suggest that transcription factors play an important role in DDR and repair; the details of how TEADs may function in DNA damage is only beginning to come to light.

The Hippo pathway is tightly networked into a variety of cellular functions, including DNA damage. For example, YAP is known to respond to DNA damage signals. Levy *et al.* have demonstrated that upon DNA damage, ATM phosphorylates c-Abl tyrosine kinase and activated c-Abl in turn phosphorylates YAP (45). This phosphorylation stabilizes YAP and promotes interaction with p73, leading to transcriptional activation of proapoptotic genes, including promyelocytic leukemia tumor suppressor. Promyelocytic leukemia tumor suppressor is then able to bind to YAP and regulates YAP stability (45, 46). In a parallel DDR pathway, ATM activation by DNA damage phosphorylates the Hippo pathway activator, RASSF1A, and stimulates the activity of mammalian STE20-like 1/2 and large tumor suppressor 1/2 to promote YAP and p73 binding (47, 48). These findings outline a tumor suppressive mechanism by which YAP-mediated protein interactions become induced by DNA damage to promote apoptosis (49). Here, we describe our observations that TEADs may also respond to DNA damage. We found that TEADs localize to DNA damage-induced nuclear foci even in cells that do not express YAP and TAZ, indicating that the function of TEADs in response to DNA damage is separate from interaction with YAP and TAZ. The details of what mechanistic role TEADs play in mediating DNA damage repair are still to be determined.

Over the past decade, the Hippo pathway proteins, YAP, TAZ, and TEADs, have been shown to be active players in tumorigenesis, metastasis, resistance to chemotherapies, and targeted immunotherapies (8, 19, 50). Inhibitors to TEAD, aimed at disrupting protein–protein interactions, are in development and advancing in the clinic. Thus, deepening our understanding of the protein interactions involving TEADs are crucial for effectively evaluating the therapeutic potential and impact of TEAD inhibitors. Our study suggests that knockdown of TEADs slows cellular proliferation and that treatment with γ-irradiation or chemotherapeutic agents can further slow cell growth and reduce DNA repair, leading to accumulation of DNA lesions. A combination of a TEAD inhibitor and chemotherapy may have a potential clinical benefit. Clearly, the TEADs–DNA damage connection deserves further scrutiny moving forward.

## Data availability

Mass spectrometry proteomics data (raw data, searched data, annotated spectra, metadata for experimental design, spectral count data) and statistical analysis result (SAINT and the enriched hallmark gene set list) have been deposited to MassIVE (https://massive.ucsd.edu/ProteoSAFe/static/massive.jsp) with the data set identifier (MSV000090760). Username: MSV000090760_reviewer, Password: reviewersonly.

Further information and requests for resources and reagents should be directed to and will be fulfilled by the Lead Contacts, Anwesha Dey: anweshad@gene.com and Jennie R. Lill: jlill@gene.com.

## Supplemental data

This article contains supplemental data (31, 32).

## Conflict of interest

A. A. is co-founder of Tango Therapeutics, Azkarra Therapeutics, Ovibio Corporation; a consultant for SPARC, Bluestar, TopoRx, ProLynx, Earli, Cura, GSK; a member of the SAB of Genentech and GLAdiator; receives grant/research support from SPARC and AstraZeneca; holds patents on the use of PARP inhibitors held jointly with AstraZeneca which he has benefited financially (and may do so in the future). All Genentech authors are employees and shareholders at Roche and have no conflict of interest.
