# Supplementary material for: TEAD Proteins Associate With DNA Repair Proteins to Facilitate Cellular Recovery From DNA Damage
Source: Mol Cell Proteomics. 2023 Jan 12;22(2):100496. doi: 10.1016/j.mcpro.2023.100496 (PMC9947421; doi:10.1016/j.mcpro.2023.100496)
Supplement: Supplementary Material [file mmc5.docx]

**Supplemental Material**

TEAD proteins associate with DNA repair proteins to facilitate cellular recovery from DNA damage

Philamer C. Calses, Victoria C. Pham, Alissa D. Guarnaccia, Erik Verschueren, Sietske T. Bakker, Benjamin Haley, Chad Liu, Matthew T. Chang, Noelyn Kljavin, Trent Hinkle, Corey Bakalarski, Meena Choi, Jianing Zou, Cuicui Yan, Xia Song, Xiaoyan Lin, Rebecca Rowntree, Alan Ashworth, Anwesha Dey, Jennie R. Lill

List of Supplemental Material Included

Table S1: Protein_Coverage_and_Search_Statistics.xlsx

Table S2: Spectra_counts.xlsx

Table S3: Saint_results.xlsx

Table S4: Compute_overlaps.xlsx

Figure S1: Comparison of replicates and treatment conditions from the WT v. mutant-NLS TEAD3 AP-MS experiments
Figure S2: Assessment of the response of YAP and TAZ to DNA damage

Figure S3: Survival assay in Detroit 562 cells upon knockdown of TEADs and treated with DNA damaging agents

Figure S4: Knockdown of TEADs does not alter the cell cycle in HeLa or U-2 OS cells

**Table S1: Protein_Coverage_and_Search_Statistics.xlsx**

The statistics for protein identification: Protein Accession, total PSM, the number of unique modified peptides, the number of covered residues, and % coverage of each protein assigned.

**Table S2: Spectra_counts.xlsx**

The spectral count data for AP-MS experiment. This table is the input for SAINTexpress.

**Table S3: Saint_results.xlsx**

The result of SAINTexpress for the AP-MS experiment is available in Table S3. This table is relevant to Figure 1D-F. Significant interactions from this table were submitted for ‘Compute overlaps’ tools against the Hallmark gene set from MsigDB.

**Table S4: Compute_overlaps.xlsx**

The enriched Hallmark gene sets from MsigDB by ‘Compute overlaps’ tool are available. Each sheet is for each cell line. FDR q-value < 0.05 was applied. This table is used for Figure 1D.


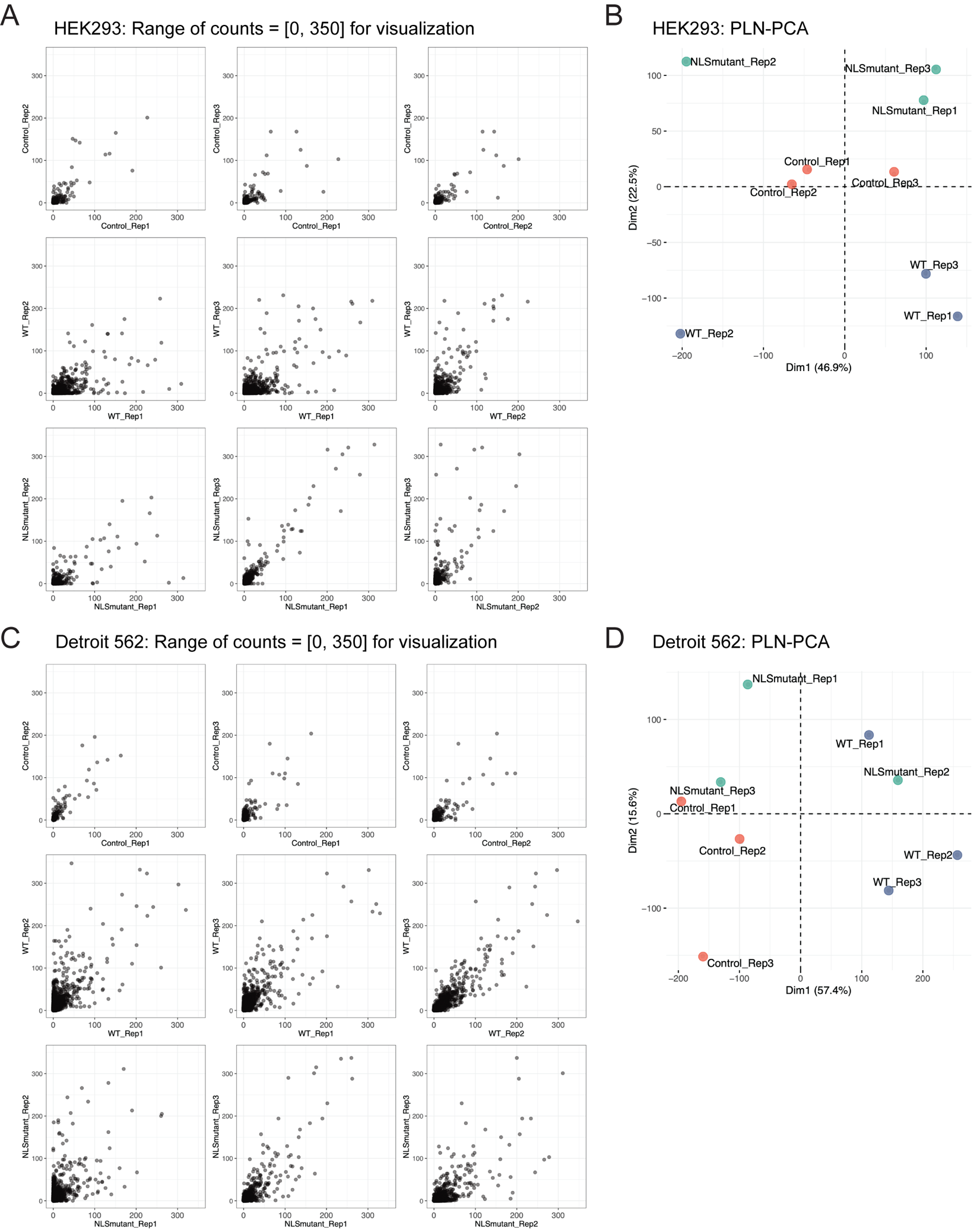
**Figure S1: Comparison of replicates and treatment conditions from the WT v. mutant-NLS TEAD3 AP-MS experiments**

(A) Scatter plots across biological replicates within the same group for spectral count data in HEK293 cells. We limited the range of counts, for both the x-axis and y-axis, to [0, 350]. This range covers 99.9% of spectral counts. (B) PLN-PCA plot for spectral count data in HEK293 cells. PLN-PCA is the dimension reduction of multivariate count data (PCA for count data) [1], PLNmodels v0.11.7, R package in CRAN was used [2]. Different groups are represented by different colors. Biological replicates form three clusters in HEK293 cells. (C) As in (A) but for Detroit 562 cells. (D) As in (B) but for Detroit 562 cells.

**
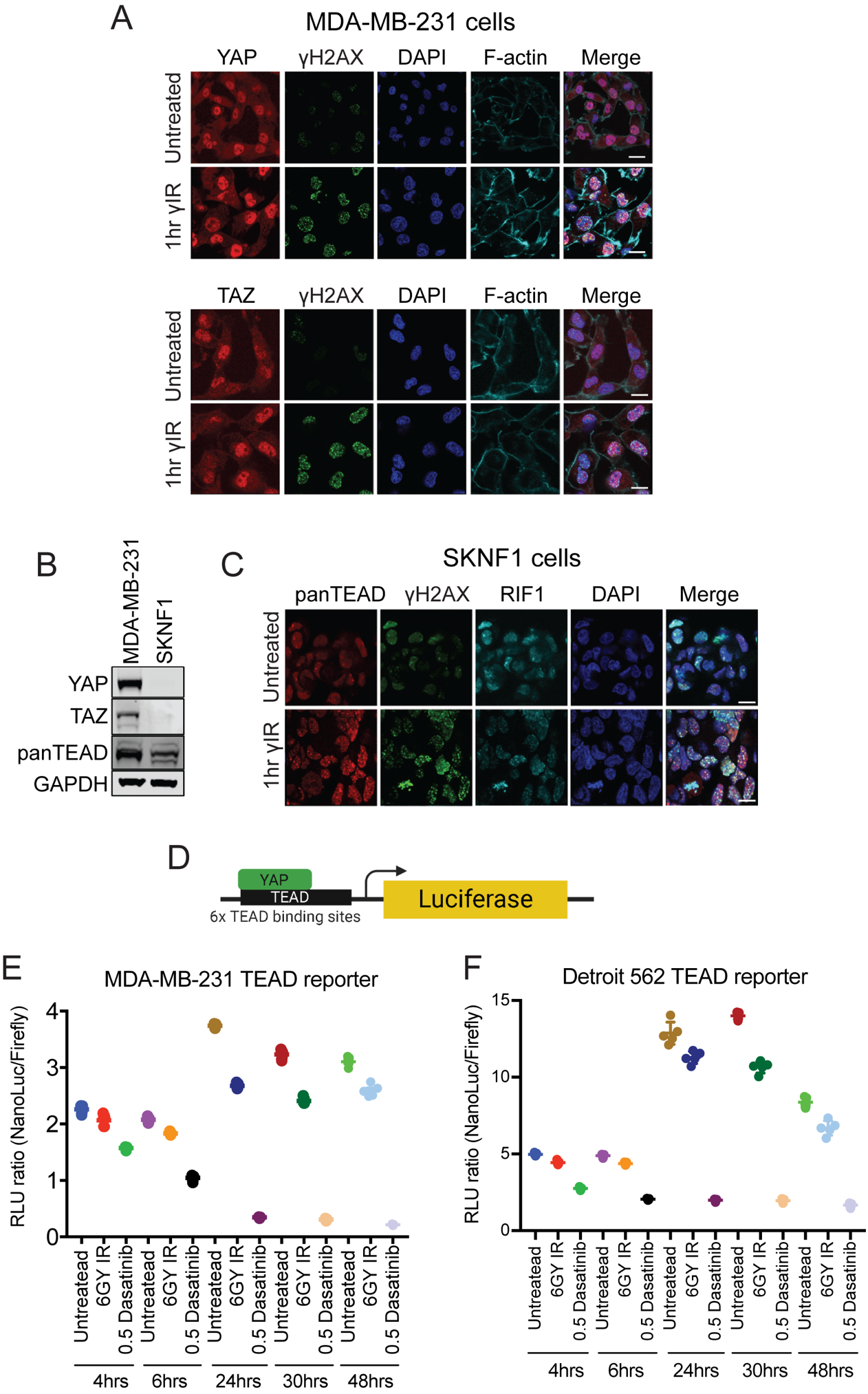
**

**Figure S2: Assessment of the response of YAP and TAZ to DNA damage**

(A) YAP and TAZ do not change localization in response to γ-irradiation. Immunofluorescence staining in MDA-MB-231 cells. Cells were untreated or treated with 6GY of γIR for one hour. F-actin marks cytosol. DAPI marks nuclei. γH2AX marks DNA damage foci. (B) SKNF1 cells do not express detectable levels of YAP and TAZ. Western blotting to compare protein levels of YAP, TAZ and TEADs in MDA-MB-231 cells and SKNF1 cells. GAPDH is a loading control. (C) Even in the absence of YAP and TAZ, TEADs colocalize with DNA damage foci. Immunofluorescence staining in SKNF1 cells. Cells were untreated or treated with 6GY of γIR for one hour. γH2AX and RIF1 mark DNA damage foci. DAPI marks nuclei. (D) Schematic of TEAD transcriptional reporter assay. Binding of transcriptionally-competent TEAD to the reporter cassette stimulates transcription of luciferase.

******Need to get info on the method detail for the transcriptional reporter assay to complete figure legend**

(E) TEAD transcriptional reporter assay in MDA-MB-231 cells. Treatment with 6 GY of γIR slightly reduces transcriptional activity while treatment with dasatinib causes a larger decrease in transcriptional activity.

Comparing treatments.

(F) TEAD transcriptional reporter assay in Detroit 562 cells.

Dasatinib is a positive control for YAP disruption.

**
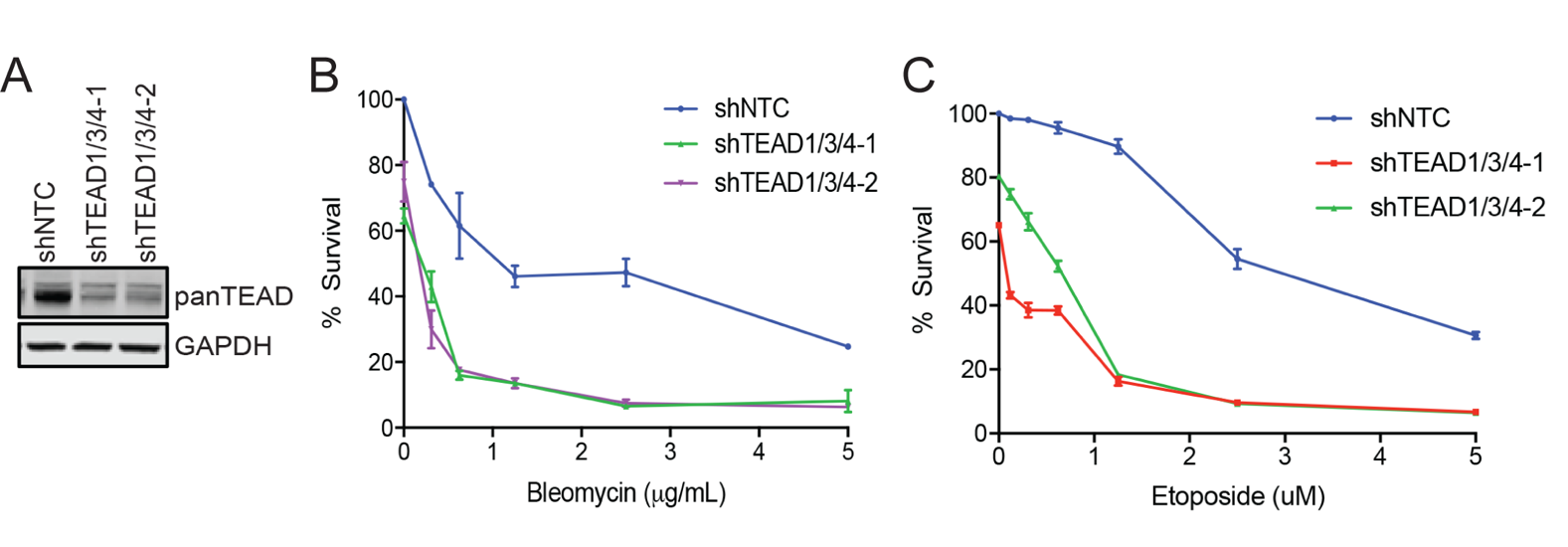
Figure S3: Survival assay in Detroit 562 cells upon knockdown of TEADs and treated with DNA damaging agents.**

(A) Western blot to evaluate knockdown of TEAD1/3/4 in Detroit 562 cells. (B) Survival assay with bleomycin. Detroit 562 cells expressing shRNAs, either non-targeting control (NTC) or targeting TEAD1/3/4, were treated with varying concentrations of Bleomycin for 24 hours. Cells were then exchanged into fresh media and incubated for five to six days before fixation and staining with crystal violet to quantify cell growth. (C) Survival assay with etoposide. Detroit 562 cells expressing shRNAs, either non-targeting control (NTC) or targeting TEAD1/3/4, were treated with varying concentrations of etoposide for 24 hours. Cells were then exchanged into fresh media and incubated for five to six days before fixation and staining with crystal violet to quantify cell growth.


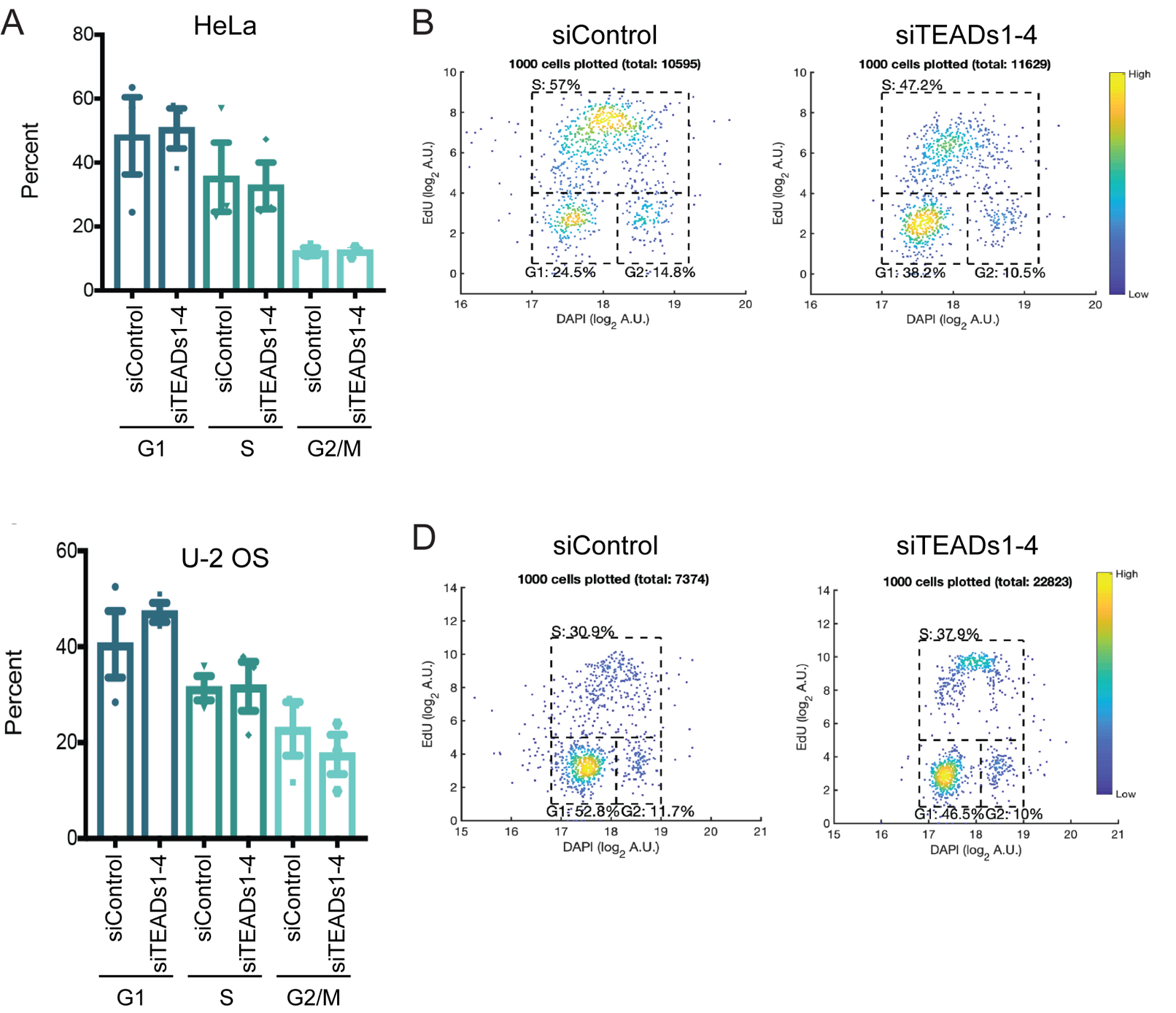
**Figure S4: Knockdown of TEADs does not alter the cell cycle in HeLa or U-2 OS cells**

(A) Cell cycle analysis of HeLa cells used in the NHEJ reporter assay in Figure 5. Cells transfected with either control siRNAs or siRNAs targeting TEAD1-4 were assayed by flow cytometry to evaluate cell cycle distributions. (B) Representative flow cytometry plots from the data quantified in (A). (C) Cell cycle analysis of U-2 OS cells used in the HR reporter assay in Figure 5. Cells transfected with either control siRNAs or siRNAs targeting TEAD1-4 were assayed by flow cytometry to evaluate cell cycle distributions. (D) Representative flow cytometry plots from the data quantified in (C).

******Need to remove citations from supplement, make sure numbering is correct, and have them in the main references section**

1. Julien Chiquet MM, Stéphane Robin (2021) The Poisson-Lognormal Model as a Versatile Framework for the Joint Analysis of Species Abundances. Frontiers in Ecology and Evolution 9. doi: 10.3389/fevo.2021.588292

2. Julien C MM, Stéphane R, Giovanni P, François G (2022) PLNmodels: Poisson Lognormal Models. R package version 0.11.7,
